# A Simple and Efficient Genetic Immunization Protocol for the Production of Highly Specific Polyclonal and Monoclonal Antibodies against the Native Form of Mammalian Proteins

**DOI:** 10.3390/ijms21197074

**Published:** 2020-09-25

**Authors:** Julie Pelletier, Hervé Agonsanou, Fabiana Manica, Elise G. Lavoie, Mabrouka Salem, Patrick Luyindula, Romuald Brice Babou Kammoe, Jean Sévigny

**Affiliations:** 1 Centre de Recherche du CHU de Québec—Université Laval, Quebec City, QC G1V 4G2, Canada; julie.pelletier@crchudequebec.ulaval.ca (J.P.); herveagonsanou@yahoo.fr (H.A.); manicafabiana@gmail.com (F.M.); elise.gaudreau-lavoie@crchudequebec.ulaval.ca (E.G.L.); mabrouka.salem@crchudequebec.ulaval.ca (M.S.); patluyind@yahoo.fr (P.L.); romuald.babou@crchudequebec.ulaval.ca (R.B.B.K.); 2 Département de Microbiologie-Infectiologie et d’Immunologie, Faculté de Médecine, Université Laval, Québec City, QC G1V 0A6, Canada

**Keywords:** immunization, antibody, protocol, guinea pig, cDNA

## Abstract

We have generated polyclonal and monoclonal antibodies by genetic immunization over the last two decades. In this paper, we present our most successful methodology acquired over these years and present the animals in which we obtained the highest rates of success. The technique presented is convenient, easy, affordable, and generates antibodies against mammalian proteins in their native form. This protocol requires neither expensive equipment, such as a gene gun, nor sophisticated techniques such as the conjugation of gold microspheres, electroporation, or surgery to inject in lymph nodes. The protocol presented uses simply the purified plasmid expressing the protein of interest under a strong promoter, which is injected at intramuscular and intradermal sites. This technique was tested in five species. Guinea pigs were the animals of choice for the production of polyclonal antibodies. Monoclonal antibodies could be generated in mice by giving, as a last injection, a suspension of transfected cells. The antibodies detected their antigens in their native forms. They were highly specific with very low non-specific background levels, as assessed by immune-blots, immunocytochemistry, immunohistochemistry and flow cytometry. We present herein a detailed and simple procedure to successfully raise specific antibodies against native proteins.

## 1. Introduction

Antibodies that detect native proteins with high specificity are essential research tools. To obtain these precious immunoglobulins, different types of antigens can be used such as synthetic peptides conjugated to a carrier. The antibodies generated against peptides often do not detect the proteins of interest in their native forms. To circumvent this limitation, purified proteins can be utilized for immunization. However, the techniques necessary to purify proteins are laborious and may denaturate the proteins of interest during the purification steps, especially transmembrane proteins. Furthermore, the level of purity necessary to raise specific antibodies is high as some of the impurities are often immunogenic. A genetic immunization approach represents an interesting alternative [1] but it generally generates sera with low titers.

In genetic immunization, the protein of interest is expressed using a plasmid containing its gene under the control of a strong enhancer-promoter such as the one from cytomegalovirus (CMV) for a high expression level. This construct is injected into the animal where it is taken up by cells and the gene of interest is expressed. As a result, the animal reacts against this “non-self” antigen and produces specific immunoglobulins. This technique has the advantage to produce an antigen 100% pure without any effort. When using the full coding sequence of a mammalian gene, the protein of interest undergoes normal post-translational modifications. Therefore, the antibodies produced are directed against the protein in a normal mammalian form.

Genetic immunization has been used in different species such as rat [2], mouse [3], monkey [4], ferret [4] and rabbit [5]. Thus far, cDNA immunization in the guinea pig was mostly used in models of infectious challenge to verify the protective effect of cDNA vaccine. Most of these cDNA vaccine protocols in the guinea pig have been established using electroporation [6,7], which requires further equipment.

In this study, we summarize our results obtained over the last two decades using cDNA injection in different animal species and we propose an optimized, easy, and convenient protocol for the generation of polyclonal as well as monoclonal antibodies. During this work, we observed that one species in particular produced polyclonal antibodies in a consistent and reproducible manner, namely the guinea pig. Monoclonal antibodies could also be obtained in mice with a similar cDNA immunization procedure, to which we added a final injection constituted of a suspension of cells transiently transfected with the protein of interest for a stronger and faster challenge.

## 2. Results

### 2.1. Polyclonal Antibodies

#### 2.1.1. Analysis of the Antibodies Produced

Over the last two decades, we have tested different immunization conditions to raise antibodies. We have tested several conditions that allowed us to identify a protocol that is very convenient and reliable to successfully raise specific antibodies excellent for research purposes. This protocol can be used by laboratories with minimal immunization experience. We will first present some of the immunization procedures that led us to the protocol that we describe at the end of this manuscript. 

The sera obtained were tested by western blot, immunocytochemistry and immunohistochemistry after the third injection and compared to their respective pre-immune sera collected immediately before the first injection. We considered that an animal serum was positive when a specific signal was obtained either in western blot, immunohistochemistry or immunocytochemistry, and absent in the pre-immune serum. The best antibodies were also tested by flow cytometry. It is noteworthy that most of the antibodies that we have generated by cDNA immunization detected the native protein with its normal disulfide bridges. Therefore, the antibodies generally did not detect the proteins of interest in western blots under reducing conditions, with either DTT or mercaptoethanol. Broadly speaking, when using this immunization technique, antisera that reacted positively in western blot under non-reducing conditions detected also efficiently the antigens by immunocytochemistry, immunohistochemistry and flow cytometry.

Most of the antibodies documented in this study can now be obtained commercially. The monoclonal and polyclonal antibodies to NTPDases, NPPs and CD73 can be obtained at ectonucleotidases-ab.com. The antibodies to Robo4, Dectin-2 and RANK were licensed to Medimabs and a different monoclonal anti-human RANK was licensed to Millipore, where they can be obtained.

#### 2.1.2. Administration Routes and Electroporation

Most of the immunization protocols were performed by intramuscular (IM) and intradermal (ID) injections, which is easy and convenient. Alternative routes of injection, some of which having been reported to elicit a stronger and more rapid immunization [8], were also tested. Administration in the subscapular area was tested in seven rabbits with different antigens and compared with ID and IM injections on 13 rabbits (see Table 1). Unfortunately, antibodies were produced only in one of those groups with the plasmid expressing mouse NTPDase2. The serum of the animal injected in the subscapular region did not give a better signal than those of the two rabbits injected at ID and IM sites. For the six other plasmids (angiomotin, Bmx/Etk, LCCP, mouse NTPDase8, RANK, RANKL), no antibody was produced either by the rabbits injected ID and IM or by those injected in the subscapular region in addition to ID and IM injections.

**Figure 1:**
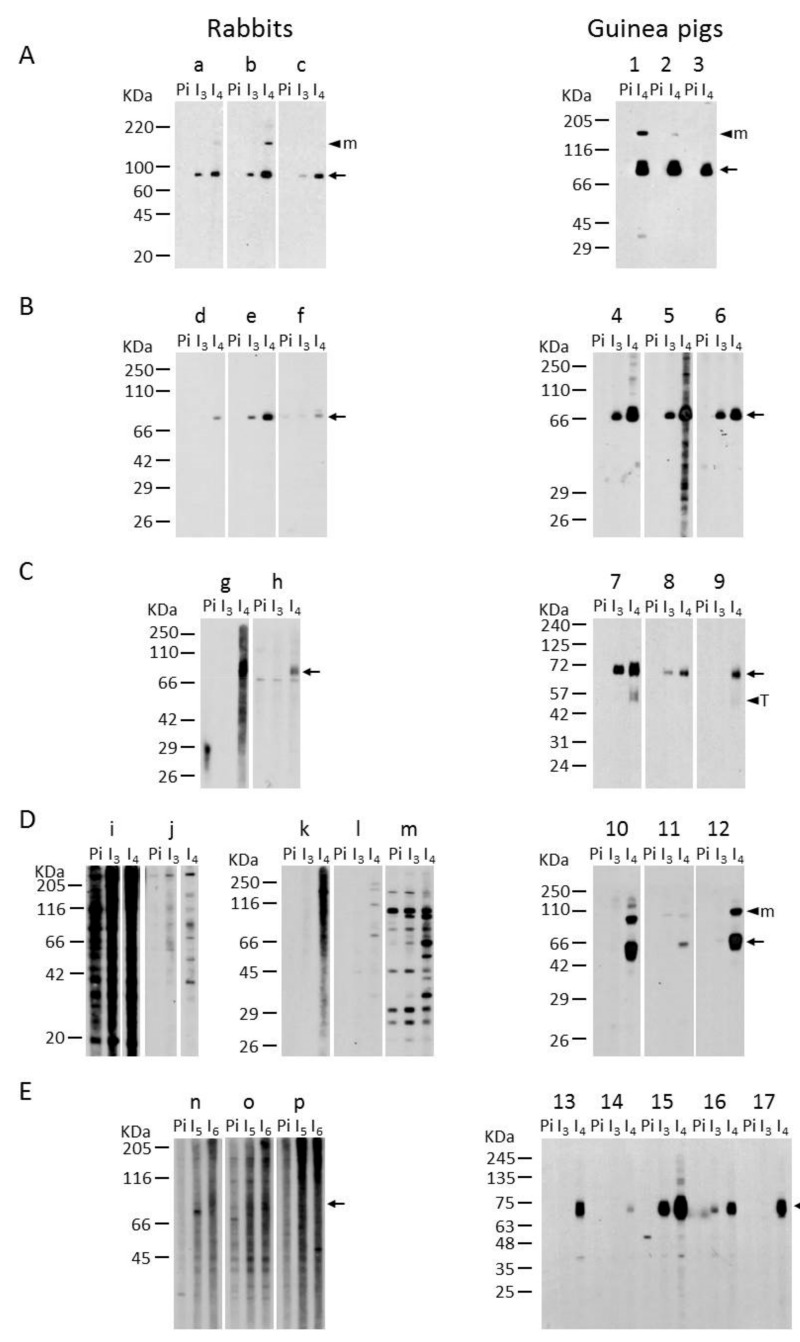
Immuno-blotting-based comparative analysis of rabbit and guinea pig antisera. Lysates for one large well from COS-7 cells or HEK 293T cells transfected with plasmids encoding human ecto-5′-nucleotidase (**A**), rat ecto-5′-nucleotidase (**B**), human NTPDase1 (**C**), mouse NTPDase3 (**D**) or mouse NTPDase8 (**E**) were subjected to electrophoresis under non-reducing conditions, electrotransferred to an Immobilon-P membrane and probed with rabbits “a” to “p” (left panels) or guinea pig “1” to “17” (right panels) antisera. The sera presented are the pre-immune (Pi) negative controls and the immune sera collected after the third (I_3_), the fourth (I_4_), the fifth (I_5_) or the sixth (I_6_) injection. Specific bands are denoted with an arrow. Multimeric (M) and truncated (T) protein forms are indicated with an arrow head. The antibodies shown were diluted 1:500 except for the rabbit “g” in panel C and rabbits “k,” “l” and “m” in panel D that were diluted 1:1000.

The injection of cDNA in lymph nodes has been reported to increase the antibody response with lower levels of cDNAs [8]. We tested the popliteal lymph node route in rabbits injected with plasmids coding for mouse NTPDase3 and rat NTPDase6. Among the rabbits injected with the mouse NTPDase3 expression plasmid, one was injected at IM and ID sites, and the other rabbit received the same amount of cDNA in ID sites as well as in both popliteal lymph nodes (see Table 1). In this assay, the serum of the rabbit that received only IM and ID injection gave a better response by flow cytometry than the serum of the rabbit that received cDNA in the popliteal lymph nodes in addition to ID injections. Meanwhile, as the sera of both of these rabbits showed very high background in western blots (Figure 1D, rabbit “j” and “i”, respectively) it is difficult to conclude whether one of those bands actually corresponds to the antigen. Similar results were obtained in another series with the plasmid encoding rat NTPDase6. In these experiments, one rabbit received only IM and ID injections and two rabbits received IM, ID and popliteal lymph node injections. The rabbits injected only at IM and ID sites gave a stronger signal by immunohistochemistry than the two rabbits injected in the popliteal lymph nodes (data not shown).

Although our limited study does not allow us to draw a conclusion on the effectiveness of these two injections routes (subscapular and popliteal lymph nodes), we obtained better responses and reliability by ID and IM immunization in the animals tested. It is noteworthy that popliteal lymph node injection requires a level of surgical technical skills that did not meet our goal of identifying an easy and convenient procedure of immunization. Furthermore, the latter injection protocols were not necessary to obtain a good antibody response. Therefore, as these injections routes were more demanding technically and that they were not improving significantly antibody production with the plasmids tested, we decided to abandon the injections of the subscapular and popliteal lymph nodes.

Electroporation was also reported to increase antibody titer [9,10]. This technique was tested on three guinea pigs and four mice injected with plasmids expressing mouse NTPDase8 and human NTPDase2, respectively. No specific antibodies were obtained using electroporation in guinea pigs while positive antisera were obtained with the same plasmid in 10 out of 11 guinea pigs when injected at ID and IM sites. In our hands, electroporation was very efficient in mice as a host where the sera of all the treated mice showed a positive immune-blotting signal with a better signal versus background ratio than the sera of the two mice that received only ID and IM injections. In agreement with the literature, electroporation induced a more rapid antibody production than that observed in response to ID or IM injections alone [9]. For the mice that received DNA-electroporation, one mouse gave a positive signal in western blot after the second injection, two mice after the third injection and the fourth mouse after the fourth injection. In comparison with the two mice that received the same plasmid at ID and IM sites, one mouse gave a positive signal after the third injection and the other mouse gave a weak positive signal after the fourth injection. Among the animals that gave a positive response with ID and IM immunizations, a positive antiserum was observed after the third injection in 10% (1 out of 10) in mice, 40% (12 out of 30) in guinea pigs and 62% (16 out of 26) in rabbits. The majority of the successful animals responded following four sets of ID and IM injections (60% (six out of 10) in mice, 91% (30 out of 33) in guinea pigs and 100% (28 out of 28) in rabbits). 

Since ID and IM injections do not require special surgical skills or specialized apparatus such as an electrical device, and as they have been shown to be very efficient routes of injection thus far, we selected those easy and convenient administration routes for our next assays.

#### 2.1.3. Immunization of Different Species

Most antibodies raised in this work were produced in rabbits. We injected 25 plasmids to 74 rabbits. The cDNA immunization in rabbits led to specific antibodies in 35 out of 74 rabbits (47%). Specific antibodies were also efficiently produced in mice, the animal of choice to generate monoclonal antibodies, with a similar ID and IM immunization protocol as that used in rabbits. Among the 28 mice injected with five different plasmids, 16 mice (57%) reacted positively. To compare this cDNA immunization procedure in different species we have also immunized three rabbits, six hamsters, six rats and 11 guinea pigs with the same antigen, a plasmid encoding mouse NTPDase8. No specific antibodies were obtained in three rabbits (Figure 1E) or in six hamsters. A positive signal was obtained in two out of six rats, but only by immuno-cytochemistry (Table 2). In contrast, 10 out of 11 guinea pigs responded to this plasmid. This prompted us to test this species further with the same immunization technique. A majority of the 54 guinea pigs injected with 17 different plasmids produced a positive and specific serum with low background (41 positives out of 54 or 76%) (Table 2).

As rabbits and guinea pigs are two species of interest for the production of polyclonal antibodies and as both of these species generated specific antibodies with our protocol, we carried out a systematic comparison between them by injecting the same six plasmids into these two species. For three of those plasmids (those expressing mouse NTPDase1, human ecto-5′-nucleotidase and rat ecto-5′-nucleotidase), antisera with similar signal intensities versus background were obtained in both species. The data are presented for human ecto-5′-nucleotidase and rat ecto-5′-nucleotidase in Figure 1A,B, respectively. On the other hand, for the three other plasmids (human NTPDase1, mouse NTPDase3 and mouse NTPDase8), rabbits failed to produce specific antibodies, while guinea pigs produced, again, highly specific antibodies with low background, as seen by immune blot (Figure 1 and data not shown). Among the 10 rabbits immunized with the latter three plasmids, five rabbits gave a weak positive signal with high background in western blot for the plasmids encoding human NTPDase1 (two/two rabbits), mouse NTPDase3 (three/five rabbits), mouse NTPDase8 (zero/three rabbits). In contrast, among the 17 guinea pigs injected with those three cDNAs, 11 guinea pigs gave a strong signal without background and five other guinea pigs showed a moderate, but clean, signal in western blot: human NTPDase1 (three/three guinea pigs), mouse NTPDase3 (three/three guinea pigs), mouse NTPDase8 (10/11 guinea pigs). Most of the antisera produced are presented in Figure 1. In general, guinea pigs generated antisera with higher titer and lower background than rabbits. 

**Figure 2:**
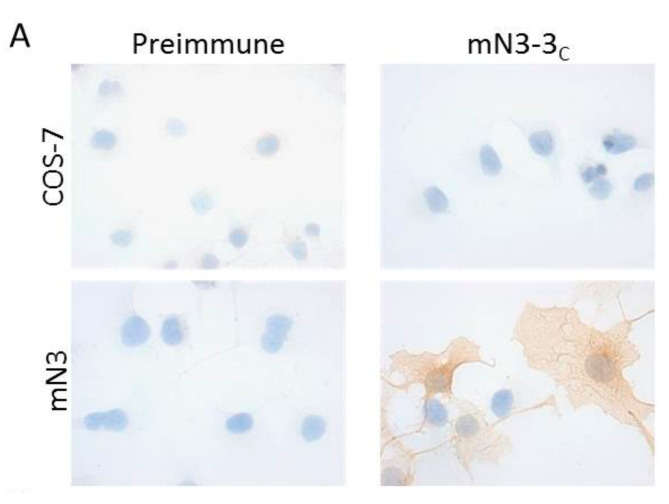

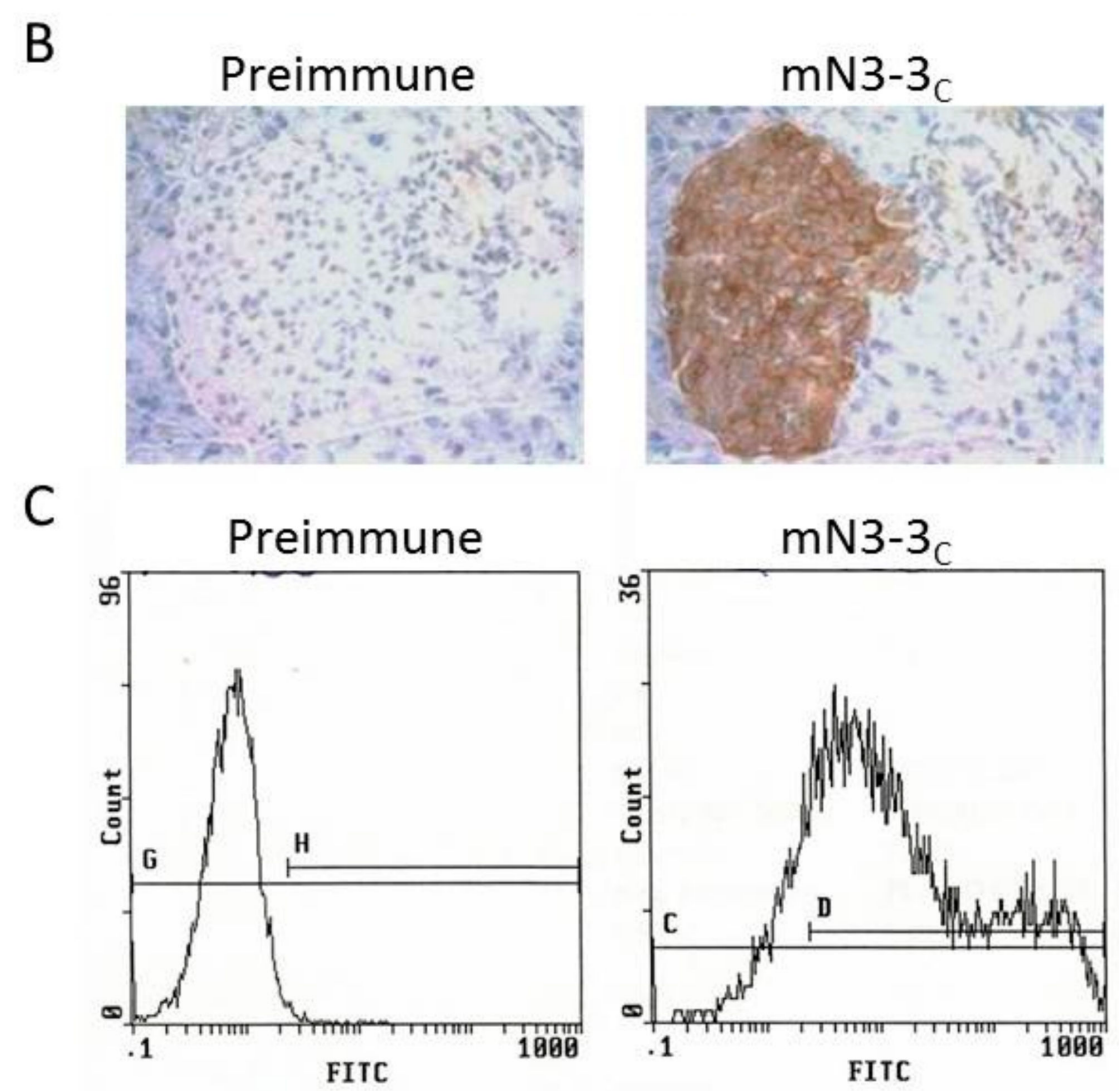
Specificity of the guinea pig anti-mouse NTPDase3 antibody mN3-3_c_. (**A**) Strong signals in immunocytochemistry of transfected COS-7 cells with a plasmid encoding mouse NTPDase3 (mN3) are only detected with antiserum mN3-3_c_ (rabbit “12” in Figure 1D). No signals are detected with the pre-immune serum or with the anti-serum on un-transfected cells (COS-7). (**B**) Immuno- histochemistry of serial sections from a mouse pancreas. The antiserum displays a positive reaction on the cells of the Langerhans islets. (**C**) Flow cytometry of transfected HEK 293T cells with a plasmid encoding mouse NTPDase3 shows a rightward shift (right panel) when compared to its pre-immune control (left panel). Nuclei were stained in blue with hematoxylin (**A**,**B**).

**Figure 3:**
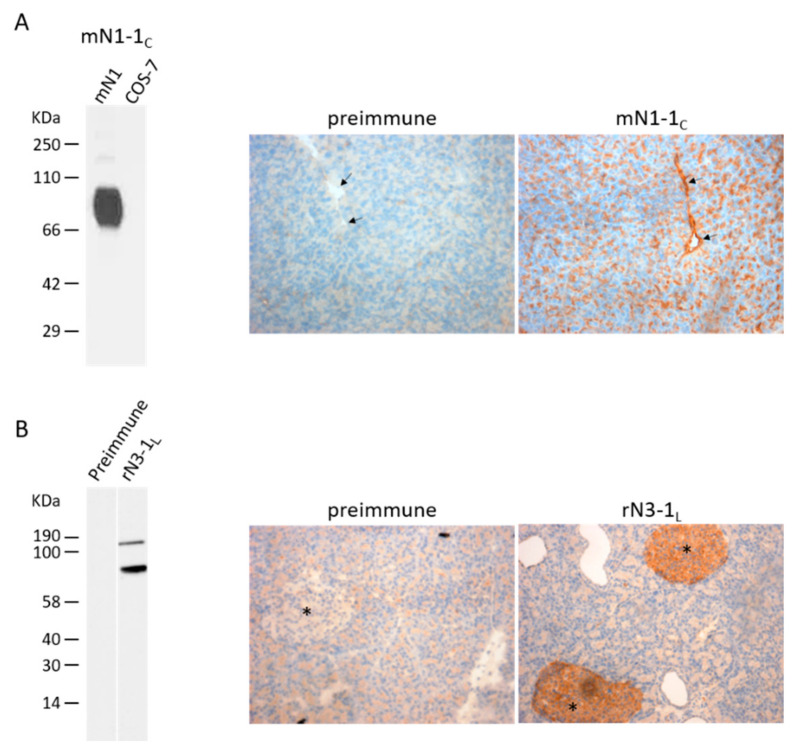
Specificity of the guinea pig anti-mouse NTPDase1 antibody mN1-1_c_ (**A**) and of the rabbit anti-rat NTPDase3 antibody rN3-1_L_ (**B**) analyzed by immunoblot (left panels) or by immunohistochemistry (right panels). A) Immunoblot of lysates from COS-7 cells transfected with plasmids encoding mouse NTPDase1 (mN1) or from untransfected COS-7 cells (COS-7). A strong reaction is observed in the transfected cell lysate only. Right panel shows immunohistochemical staining of a mouse pancreas section incubated with the antiserum mN1-1_c_ or with the pre-immune serum. The antiserum displays a strong positive reaction on blood vessels shown by arrows and on zymogen granules in pancreatic acinar cells. (**B**) Immunoblot of lysates from COS-7 cells transfected with plasmids encoding rat NTPDase3 incubated with the antiserum rN3-1_l_ or with its pre-immune serum shows a strong reaction only with the antiserum. NTPDase3 is detected as both a monomer and a dimer. Right panels show immunohistochemical staining of rat pancreas sections incubated with the antiserum or the pre-immune serum. The antiserum displays a positive reaction on Langerhans islet cells which are indicated by a star. Nuclei were stained in blue with hematoxylin in immunohistochemistry. The antisera were diluted 1:500 except for mN1-1_c_ in the immunoblot (**A**) that was diluted 1:5000.

All antisera were also tested by immunocytochemistry and immunohistochemistry, and the positive ones by flow cytometry. Nearly all of the antisera that gave a positive signal by western blot in non-reducing conditions also detected the protein of interest by immunocytochemistry, immunohistochemistry and flow cytometry, and vice versa. The antiserum mN3-3_c_ against mouse NTPDase3 (rabbit “12” in Figure 1D) is presented as an example in Figure 2 and the specificity for the antisera mN1-1_c_ and rN3-1_L_ are presented in Figure 3.

### 2.2. Mouse Monoclonal Antibodies

We also tested the ability of the above-described cDNA-based immunization technique to generate monoclonal antibodies in the mouse. To produce monoclonal antibodies, we first tested if the ID and IM injections could induce a sufficiently strong immune response to subsequently isolate monoclonal antibodies. The ID and IM injection procedures, with or without electroporation, were unsuccessful. Although electroporation have previously been reported as an immunization technique to produce monoclonal antibodies [11], we did not succeed to get a hybridoma producing a desired immunoglobulin after four to seven injections of cDNA coding for the protein of interest in two rats and two mice. To increase the titers and to get better chances in obtaining positive hybridomas, we injected HEK 293T cells transiently overexpressing human NTPDase2 to a mouse that was injected with the same plasmid encoding human NTPDase2 and that was treated by electroporation. The injection of transfected cells was done with cells never seen by the animal and the ELISA screening assay was performed with another transfected cells lines, COS-7, to eliminate false positives as much as possible. This led to the production of four hybridomas producing excellent monoclonal antibodies [12]. We then tested if the injections of HEK 293T that produces the protein of interest would also be successful in a mouse that received only IM and ID injections. From seven fusions performed in these conditions for human NTPDase1, human NTPDase2, human NTPDase3, human NTPDase8 and human Rank, three fusion procedures allowed us to obtain hybridomas that produced monoclonal antibodies against human NTPDase3 [13,14], human NTPDase8 [15] and human RANK. These data suggest that ID and IM injections, without electroporation, can be sufficient to prime the animals, and that a final injection with the recombinant protein expressed by transfected cells induces a rapid and high production of activated B cells suitable for the isolation of specific hybridomas.

### 2.3. Protocol Proposed

The above data led us to propose the protocol in Table 3 that we now routinely use for rabbits, guinea pigs and mice. The plasmid containing the protein of interest is injected at ID sites and at IM sites at an interval of 2 to 3 weeks for the first three injections and then at 8 to 10 weeks intervals for the two subsequent injections. For guinea pigs and rabbits, we suggest testing a serum sample after the third and fourth injection to identify the good responders. If a strong immunoreaction is observed after the third injection, rabbits and guinea pigs should be exsanguinated after a fourth and final immunization. If guinea pigs and rabbits do not respond after the fourth injection, they should be sacrificed as they will unlikely respond with more injections. On the other hand, five cDNA immunization procedures should be done in mice before selecting the best one for the final injection before fusion with myeloma cells.

As reliability to get antibodies is better in the guinea pig species, we suggest a lower number of guinea pigs (two or three) than rabbits (three to five) or mice (five to 10). Indeed, we obtained similar excellent antisera in all responding guinea pigs tested, which was not the case for the two other species. As mice exhibited a highly variable response from animal to animal (57% of the mice responded positively) while not requiring large amounts of plasmids, and having low housing costs, it would be wise to use two cages of mice (eight–10 mice) in order to get a better probability in obtaining a high responder in the first four or five injections to be selected for the final injection with transfected cells and for fusion with myeloma cells. The final injection can be done with 10 to 18 million HEK 293T cells transfected with the same plasmid used for the ID and IM injections. We recommend collecting the spleen three days later to perform the fusion with SP2/0 myelomas cells.

## 3. Discussion

We produced in the last two decades several antibodies using cDNA immunization techniques. We tested different strategies that finally led us to propose the protocol presented in Table 3 that we now use routinely in guinea pigs. When larger amount of serum or when antibodies from different species are necessary, or in the rare case where guinea pigs do not respond, we also use rabbits. Obviously, when a monoclonal antibody is necessary, we do it in mice with a similar protocol, also presented in Table 3, with the difference that we perform a final injection with the recombinant proteins to elicit a faster and stronger response using an expression cell system that has never been seen by the immunized animals. Indeed, the injection of HEK 293T cells expressing the protein of interest three days before the fusion procedure was more successful to obtain clones expressing the monoclonal antibodies of interest than injecting only DNA for all immunization steps. Other groups have also come to the same conclusion [16,17]. Obviously, this last injection would be inappropriate when generating polyclonal antibodies as the background would be expected to increase, which is not an issue when producing monoclonal antibodies as the desired B cells are cloned. Along the same line, it may be advantageous to transfect a mouse cell line for the final injection to reduce the number of false positive clones, but this might trigger a weaker immunological response and cause lower chances in getting hybridomas. This could be a subject for future improvement.

Guinea pigs are often the only species that we now immunize as they are high responders to this protocol and the response is generally similar from one animal to the other, which was not the case with mice and rabbits. There is therefore no need to inject five or six guinea pigs with our technique, as is often suggested in immunization protocols for most animals. Another advantage of using guinea pigs over rabbits is the much lower amount of DNA necessary (three–four times less) to trigger an antibody response. Depending of the animal facilities, housing costs for guinea pigs are also generally less expensive than rabbits.

Antibody response has been reported to be increased when injecting the cDNA in lymph nodes, or when using electroporation, but as plasmids can be easily produced in large amount, and with an extremely high purity, it is much easier to produce more plasmids to inject at ID and IM sites rather than to inject in the lymph nodes to save some plasmids. This is especially important when the surgery skills are not at hand. The same situation applies when comparing the technique that we present in Table 3 with other techniques using expensive technologies such as the injection of gold or tungsten conjugated particles with a gene gun. As the technique presented in Table 3 is efficient and easy to perform, there is no need to buy expensive equipment or to use unnecessarily sophisticated techniques.

cDNA immunization technique, as presented here, elicited an antibody response that reacted against the antigen in its native form. This is of interest when using plasmids encoding mammalian protein antigens to immunize guinea pigs, rabbits or mice that will perform mammalian post-translational modifications, although they can differ slightly between mammalian species. As post-translational modifications are different in non-mammalian species, the protocol proposed here might not be as efficient to raise antibodies against antigens in their native forms when they originate from non-mammalian sources, such as for antigens from prokaryotes or plants. This will depend on the epitope(s) selected by the host animal. Another issue, especially when using non mammalian antigens, is to make sure that the expression level of the gene is adequate in the host animal. This can be corrected or ameliorated by codon optimization of the gene of interest. In agreement with this idea, an increased antibody response was reported by a few groups after optimizing the codon sequences of some HIV genes [18,19,20,21]. Post-translational modifications and codon optimization could therefore represent a subject for future amelioration of cDNA immunization.

Another point to consider when using this technique is the protein location after its expression from the plasmid. Indeed, as we did in this paper, cDNA immunization has mostly been used to produce antibodies against transmembrane proteins. Several groups have also reported some success in generating antibodies against intracellular and secreted proteins by gene gun [22,23] or electroporation [24,25,26]. Efficiency to produce antibodies against an intracellular protein by injecting cDNA at ID or IM sites differs from group to group. Few studies have compared the same intracellular protein with or without a signal peptide to mediate protein secretion. In one of these studies, a plasmid with or without such a signal sequence was injected IM and a similar antibody response was reported for both plasmids [27]. Other studies reported a better efficiency when a signal sequence was added to a gene coding for an intracellular antigen [28,29]. In the results presented here, the majority of the plasmids encoded a transmembrane protein which led to specific antibody in 80% (41 out of 51) in guinea pigs, 60% (33 out of 55) in rabbits and 57% (16 out of 28) in mice. In our study, we did not succeed to raise antibodies in rabbits (zero out of 11) injected with a plasmid coding for an intracellular protein. The immune responses to an antigen encoded by plasmids coding for secreted proteins were slightly stronger. Although we did not succeed in guinea pigs (zero out of three) a few rabbits (two out of eight) produced antibodies against a secreted protein. Another alternative to get antibodies to soluble proteins is to modify the protein by adding a transmembrane domain to direct the protein to the external surface of the plasma membrane. We tested this idea with one plasmid, which successfully produced antibodies in one out of five mice (unpublished data in collaboration with Dr J. Fietto, Universidade federal de Viçosa, Brazil). This is another avenue of study to make cDNA immunization more efficient for more protein types.

As there is no immunization technique that works all the time, when should we stop to immunize an animal with our protocol presented on Table 3? We observed that when there was no positive signal after the fourth injection in guinea pigs and in rabbits, there was limited chance to obtain a good antiserum by doing additional injections. Indeed, the few times that a fifth injection was positive for the guinea pigs, the titer was too low to be useful for a research purpose. Therefore, we now sacrifice all guinea pigs and rabbits that do not give a good response in the first four immunizations. On the other hand, injecting a good responder too many times might not be beneficial, and on the contrary, the antibody response might decrease and the background signals may increase. Accordingly, when a rabbit or a guinea pig responds well at the third injection, it should be exsanguinated after the fourth injection. Our observations were different in mice as we observed some mice that did not react at the fourth injection but gave an interesting response after the fifth injection. But as this was seen only in a few mice (four out of 10) with the same plasmid, we cannot draw any definitive conclusions at the moment concerning this point. Nevertheless, it might be safer to try a fifth DNA injection before sacrificing mice. Indeed, according to our results, mice often required five cDNA injections to get a strong positive signal.

## 4. Materials and Methods 

### 4.1. Materials

Hypoxanthine-aminopterin-thymidine solution, hypoxanthine-thymidine solution, poly-ethylene glycol and bovine serum albumin were purchased from Sigma-Aldrich (Oakville, ON, Canada). Dulbecco’s modified Eagle’s medium and Lipofectamine were obtained from Life Technologies (Burlington, ON, Canada). Fetal bovine serum (FBS), phosphate-buffered saline (PBS), antibiotic/antimycotic and HCell-100 hybridomas media were purchased from Wisent (St-Bruno, QC, Canada). Secondary antibodies conjugated to horseradish peroxidase (HRP) were obtained from: goat anti-mouse IgG (H + L) from Jackson ImmunoResearch Laboratories Inc. (West Grove, PA, USA), goat anti-guinea pig from Santa Cruz Biotechnology (Dallas, TX, USA), donkey anti-rabbit from GE Healthcare Life Sciences (Baie d’Urfe, QC, Canada). The secondary goat anti-guinea pig antibody conjugated to biotin was from Jackson ImmunoResearch Laboratories Inc. (West Grove, PA, USA). Enhanced K-Blue^®^ Substrate was from Neogen Corporation (Lansing, MI, USA). 

### 4.2. Animals

Female New Zealand rabbits (4 months), Hartley guinea pigs (6–7 weeks), BALB/c mice (4–6 weeks), Sprague-Dawley rats (7 weeks), LVG Golden Syrian hamsters (7 weeks), were obtained from Charles River Laboratories (St-Constant, QC, Canada). For two antigen series, rabbits (four) were 9–10 weeks old. All procedures were approved by the Canadian Council on Animal Care and the Université Laval Animal Welfare Committee (protocol number: 2001-120; 2004-179; 2007-124; 2010-102; 2013-108; 2017-109). 

### 4.3. Immunization

Complementary DNA (cDNA) was prepared using Endofree plasmid kits from Qiagen (Toronto, ON, Canada) and diluted in PBS 0.8× at a concentration range of 0.5–1 mg/mL. Intradermal (ID) or intramuscular (IM) injections were performed in the dorsal skin or in a thigh, respectively. Two groups of rabbits received the cDNA in the popliteal lymph node(s). This injection route was performed once during the injection schedule and the rabbits received ID and IM injections for the other injections. In one of these groups, rabbits received DNA coding for mouse NTPDase3 in popliteal lymph nodes of both legs at the second injection in addition to ID injections. In the second group, rabbits received DNA coding for rat NTPDase6 in a popliteal lymph node of one leg at the first injection, in addition to ID and IM injection. Specific information related to the number of sites, amount injected and injection schedule is reported in Table 1. Each animal received at least five series of immunization to assure adequate priming. In some cases, animal received up to nine series of immunization. 

### 4.4. Electroporation 

Mice and guinea pigs received DNA followed by an electroporation as previously described [9,12]. Briefly, an IM injection of DNA diluted in Hank’s balanced salt solution or in PBS, for the mouse or the guinea pig, respectively, was made in the tibialis anterior. After application of an electrode cream on the skin, electroporation was carried out at the site of DNA injection with two electrode plates connected to an electroporator according to the following parameters: seven pulses of 100 V.cm^−1^ and a duration of 20 ms.

### 4.5. Blood Collection

Blood was collected before the first injection and 11 to 15 days after the second injection when an electroporation was performed, or after each injection after the third one. Blood was collected in spray-coated silica tubes with or without gel separator or in regular microcentrifuge tubes. Tubes were let in the upright position for 1–2 h at room temperature (RT) to allow clot formation, and were then centrifuged at 1500× *g* for 10–30 min depending on the blood volume. A final concentration of 10% glycerol was added to the serum which was then aliquoted and stored at −80 °C until needed.

### 4.6. Monoclonal Antibodies Raised in Mice 

After four to six DNA injections with or without electroporation, as described in Table 1, some mice received a final injection of DNA, and others an injection of human embryonic kidney (HEK 293T) transfected cells. These cells were transfected with Lipofectamine as previously described [30] with the same plasmid used for the previous immunization procedures. Then, 2 days after transfection, the cells were washed twice with PBS and the cells were detached by a 2–5 min incubation at 37 °C in a citric saline solution (135 mM potassium chloride, 15 mM sodium citrate). Cells were centrifuged, resuspended in PBS and counted. The mouse received a final intraperitoneal injection of 10 to 18 million of transfected HEK 293T. The fusion procedure was performed either 6–7 days, or 3 days, after this final injection of DNA, or the cell suspension, respectively. Blood and spleen cells were collected after this final injection and fusion with SP2/0 cells was done as described [13]. The supernatant was screened by ELISA and the positive hybridomas were cloned by limiting dilution. In a few assays, a hybridomas optimized medium, Hcell-100, was used for the clone isolation procedures. The produced immunoglobulins were purified on Protein A Sepharose CL-4B column as described [13]. 

### 4.7. ELISA

ELISA was performed as previously described [15]. In brief, protein extract (500 ng per well) from untransfected African green monkey kidney (COS-7) cells or transiently transfected with the plasmid used for immunization diluted in PBS was distributed in a 96 well ELISA plate and incubated overnight at 4 °C. The wells were then washed with PBS-Tween 0.05% (PBS-T) and incubated for 1 h at 37 °C in a blocking solution (0.5% bovine serum albumin diluted in PBS-T). After washing, the supernatant from each hybridoma was added to a well and incubated for 2 h at RT, followed by four washing steps. Then, a goat anti-mouse IgG (H + L)-HRP (1:2500) diluted in the blocking solution was incubated for 2 h at RT, followed by four washing steps. The Enhanced K-Blue^®^ Substrate was then added for 15 min and the reaction was stopped by the addition of an equal volume of 2 N sulphuric acid. The absorbance at 450 nm was then recorded. 

### 4.8. Isotyping

Mouse Immunoglobulin Isotyping ELISA Kit (BD Bioscience, Mississauga, ON, Canada) was used to determine the isotypes produced by the hybridomas, according to the manufacturer’s instruction and as previously described [15]. In brief, monoclonal rat anti-mouse IgG1, IgG2a, IgG2b, IgG3, IgA and IgM were coated O/N in 96-well plates. A blocking treatment was performed after washing steps, and then each monoclonal antibody was transferred to the wells. After four washing steps, rat anti-mouse Igs conjugated to HRP was added to each well, and revealed with a substrate provided in the kit. The plate was then read at 450 nm.

### 4.9. Western Blot

COS-7 cells and HEK 293T cells were cultured and transiently transfected with the indicated cDNA construct as described previously [30]. For western blot assays, lysates from transfected COS-7 cells or HEK 293T cells were resuspended in NuPAGE LDS sample buffer, separated on NuPAGE 4–12% Bis-Tris gels under non-reduced conditions, and transferred to an Immobilon-P membrane (Millipore, Bedford, MA, USA) by electroblotting according to the manufacturer’s recommendation and as previously described [15]. Membranes were then blocked with 2.5% non-fat milk in PBS containing 0.15% Tween20 (pH 7.4) O/N at 4°C and subsequently probed with the primary antibodies using the Mini-Protean II multiscreen apparatus (Bio-Rad Laboratories Ltd., Mississauga, ON, Canada) in which 20 antibodies can be tested on one gel. Appropriate secondary HRP-conjugated antibodies were used, and the membranes developed with the western Lightning^TM^ Plus-ECL system (PerkinElmer Life and Analytical Sciences, Waltham, MA, USA). 

### 4.10. Immunocytochemistry and Immunohistochemistry

Immunocytochemistry and immunohistochemistry were performed as previously described [15]. Tissues were frozen in Tissue-Tek^®^O.C.T.^TM^ Compound (Sakura Finetek, Torrance, CA, USA). COS-7 cells or tissue sections (6 μm thick) were fixed with cold acetone and 10% phosphate-buffered formalin (Fisher Scientific, Ottawa, ON, Canada) (19:1) and blocked in a PBS solution containing 7% normal goat serum for 30 min. COS-7 cells and tissue sections were incubated with the indicated primary antibody at 4 °C or pre-immune serum as a negative control. COS-7 cells and tissue sections were then treated with 0.15% H_2_O_2_ in PBS for 10 min to inactivate endogenous peroxidase, and with an avidin/biotin solution (Avidin/Biotin Blocking kit; Vector Laboratories, Burlington, ON, Canada) to prevent non-specific staining due to endogenous biotin. This step was followed by incubation with an appropriate biotin-conjugated secondary antibody at a dilution of 1:1000. The avidin–biotinylated HRP complex (VectaStain Elite ABC kit; Vector Laboratories) was added to optimize the reaction. Peroxidase activity was revealed with DAB as the substrate. Nuclei were counterstained with aqueous hematoxylin (Biomeda, Foster City, CA, USA) in accordance with the manufacturer’s instructions.

### 4.11. Flow Cytometry

Flow cytometry was performed as previously described [15]. Briefly, HEK 293T cells transfected with the plasmid expressing the antigen were detached from the plates with a citric saline solution (135 mM potassium chloride, 15 mM sodium citrate). Cells were washed with an ice-cold PBS solution containing 1% FBS and 0.1% NaN_3_ (fluorescence-activated cell sorting (FACS) buffer) followed by incubation with the immune serum or the pre-immune serum in FACS buffer for 1 h. After washes with FACS buffer solution, the cells were incubated with the appropriate FITC-conjugated secondary antibody (Jackson ImmunoResearch Laboratories Inc., West Grove, PA, USA) for 30 min on ice, washed with FACS buffer, and analyzed by flow cytometry (BD LSR II, BD Biosciences, San Jose, CA USA).

## 5. Conclusions

In this paper we propose a detailed protocol for cDNA immunization in guinea pigs and rabbits for the production of polyclonal antibodies and in mice for the generation of monoclonal antibodies. The procedure does not require any sophisticated technical expertise nor any specialized equipment. This protocol works especially well in guinea pigs which produce antisera with high specificity and extremely low background in a consistent manner, allowing the use of fewer animals per antigen to reduce effective costs, especially when comparing with larger animals. To generate monoclonal antibodies in mice, we propose to perform the last injection with a suspension of cells expressing the recombinant protein at a high level.

## Figures and Tables

**Table 1:** Immunization protocol summary.

Species	Number of Animals	Number of Antigens Tested	Administration Route	Number of Site × Volume per Site	DNA Injected per Immunization (µg)	Injection Intervals (Weeks Between Each Injection)	
						Injection 1 to 3	Injection 3 to 6
Rabbit	64	25	ID IM	6−10 × 50 µL 2−4 × 125−250 µL	300–800	2–4	8–17^a^
	7	7	ID IM SS	6−8 × 50 µL 2 × 150−175 µL 1 × 350 µL	625–1000		
	3	2	ID Pop ± IM	6−8 × 50 µL 1−2 × 150 µl 1 × 250 µl	800		
Guinea pig	50	17	ID IM	2 × 50 µl 1 × 100 µl	125–200	1.5–4	7–16
	2	1	ID	4 × 50 µL			
	2	1	IM	2 × 100 µL			
	3	1	IM + EP	1 × 100 µL	100–400	5–7	8
Mouse	20	6	ID IM	2 × 25 µL 1 × 50 µL	100	2–3	8–12
	4	1	IM	2 × 50 µL			
	4	1	IM + EP	1 × 30 µL	60	3–8	7–11
Rat	2	1	ID IM	2 × 50 µL 1 × 100 µL	200	2	8–16
	2	1	ID	4 × 50 µL			
	2	1	IM	2 × 100 µL	125–200		
Hamster	2	1	ID IM	2 × 25 µL 1 × 50 µL	100	2	10
	2	1	ID	4 × 25 µL			
	2	1	IM	2 × 50 µL			

EP: electroporation; ID: intradermal; IM: intramuscular; Pop: Popliteal; SS: Subscapular. ^a^: for the mouse NTPDase8 antigen injected to three rabbits a total of nine injections were performed at an interval of 4 to 10 weeks for the last three injections.

**Table 2:** Polyclonal antibodies raised.

Species	Number of Plasmids Tested	Number of Animals Immunized	Responding Animals (number, %)
Rabbit	25	74	35, 47%
Guinea pig	17	54	41, 76%
Mouse	5	28	16, 57%
Rat	1	6	2, 33% *
Hamster	1	6	0

Compilation of animal antisera that were considered positive when a specific signal was obtained in either western blot, immunohistochemistry or immunocytochemistry, and absent in the pre-immune serum. * A positive signal could be detected by immunocytochemistry but not by western blot.

**Table 3:** Proposed protocol to raise polyclonal and monoclonal antibodies by cDNA immunization.

Species	Route	Number of Sites × Volume per Site	DNA Concentration (mg/mL)	DNA Injected per Immunization (µg)	Injection Intervals (Weeks between Each Injection)		Blood Collection (Days after Injection)	Spleen Collection (Days after Transfected Cell Injection *)	Number of Animals per Antigen
					Injection 1 to 3	Injection 3 to 5 ^#^			
Rabbit	ID IM	6–10 × 50 µL 2–4 × 125–250 µL	0.5–0.8	500–800 ^&^	2–3	8 ^¶^	13–14	N/A	3–5
Guinea pig	ID IM	2 × 50 µL 1 × 100 µL	1	200	2–3	8 ^¶^	12–13	N/A	2–3
Mouse	ID IM	2 × 25 µL 1 × 50 µL	1	100	2–3	7^¶^	12–13	3	5–10

The final injection before the fusion with SP2/0 cells should be done with 10 to 18 million HEK 293T transfected cells using a high efficiency transfection system. Other related cell lines can be used for transfection. # If a strong immunoreaction is observed after the third injection, rabbits and guinea pigs should be exsanguinated after a fourth and final immunization. ^&^ A lower amount of plasmids (650 ± 50 µg) is suggested for the first four injections, and a higher amount (800 µg) for the last injection. ^¶^ As the animals remain primed for several weeks to months, the intervals between the last injections can be longer.

## Abbreviations

cDNA	Complementary DNA
COS-7	African green monkey kidney cells
DAB	3, 3′ diaminobenzidine
ELISA	Enzyme-linked immunosorbent assay
FBS	Fetal bovine serum
FRQS	Fonds de Recherche du Québec – Santé
HEK 293T	Human embryonic kidney 293T cells
HRP	Horseradish peroxidase
ID	Intradermal
IM	Intramuscular
PBS	Phosphate-buffered saline
PBS-T	PBS-Tween
Pi	Pre-immune serum
RT	Room temperature
NSERC	Natural Sciences and Engineering Research Council of Canada
